# A Treatise on the Practice of Medicine

**Published:** 1852-09

**Authors:** 


### REVIEW.

ART. II.—*A Treatise on the Practice of Medicine.* By GEORGE B. WOOD, M. D., Professor of the Theory and Practice of Medicine in the University of Pennsylvania; President of the College of Physicians of Philadelphia, &c. &c. Third Edition. 2 vols. Philadelphia: Lippincott, Grambo & Co. 1852.

Practical works on medicine are ever likely to be more popular with practitioners of the healing art, than those of a theoretical character, and in the ratio that medical books, purporting to be of a practical character, approach what they assume to be, in that proportion are they likely to win a present, and to maintain an enduring popularity. Indeed, much is likely to be forgiven in a practical treatise, if it approaches to a redemption of its promises. When a medical reader finds himself under the guidance of a cool, calm, dispassionate observer of disease, to whom extensive opportunities have been given for grappling with disease; when these have been faithfully used; when such an observer recognises the noble mission given to him, and makes an honest record of what he has seen, we repeat, when a medical reader finds himself in the hands of such an instructor, his confidence is won, and his mind jumps at the opportunity for instruction. Authors of practical works in medicine often forget their high vocation, and instead of making their books the medium of imparting useful knowledge, they crowd it with incidents of personal achievements with means, that are rarely *as*

successful in the hands of others. Another class of these authors are the merest hobby-riders. They assume that all the world was blind up to the time of their advent, and they ride their hobby greatly to their own satisfaction.

There are few responsibilities in author-craft that equal those assumed by the medical writer, especially if his position be such as to lend weight to his views He is capable of inflicting no ordinary amount of evil upon the human family. Those who are familiar with the history of Galen, Celsus, Boerhaave, and Rush, are prepared to estimate the force of our remark. But when a medical writer makes truth, and truth alone the goal of his wishes, and uses his position to give force and efficiency to that great object, he deserves, and should receive the confidence and esteem of his professional brethren. Such an author we find in the writer of the work before us. He has long enjoyed a distinguished rank in his profession, and has never lost sight of what was due from that rank. He has constantly labored to advance the interests of his profession by every laudable method, and his great and useful labors have given him the respect, esteem and confidence of his brethren. They have stamped the seal of their approbation upon the labors of Professor Wood, in the field embraced in this work. In 1847, the first edition appeared, and in two years after, a new one was demanded, because the first was exhausted. In 1849, another edition was published, and though twice the size of the first, it is already exhausted, and a third is now needed. These facts are scarcely more complimentary to the labors of Professor Wood, than to the good sense, true taste, and proper appreciation of medical readers. The work richly deserves just such triumphs as it has won, and we hope it will long continue to enjoy the regard of the medical profession. Its popularity shows the discriminating studies of medical practitioners, and that the author who aims at a high mark may rest assured he wins everything by hitting it.

We have indicated some of the foundations on which books of this character should rest, and Professor Wood shows that these foundations belong to the structure he has erected. He says: "Having been engaged, for nearly thirty years, in public and private practice, and, during that time, devoted an almost exclusive attention to the study of diseases and their remedies, he has accumulated facts, and formed opinions, which have been long soliciting expression, with an urgency to which he has at length yielded, though unfeignedly distrustful of their sufficient value."

These are most excellent reasons for such a work as this of Professor Wood's, and a large portion of it will make the reader thankful that any reasons whatever, induced the publication. It is not to be expected that any work on medicine can be suited to every meridian. The influence of occupation, of habits of life, of methods of sustenance, of climate, and of race, is too well known to require much in the way of illustration. Let us take a simple example. The profession is largely indebted to Marshall Hall for his valuable work on blood-letting. That work contains a large amount of honest observation, most honestly recorded, but the Western or Southern practitioner, in this country, who should undertake to make it his guide, would condemn many a patient to weeks of unnecessary suffering, and commit more grave offences. Marshall Hall made his observations in the haunts of the crowded, ill-fed population of Manchester, where the sins of miserable ventilation alone would be sufficient to deter a medical man from the use of the lancet. It is quite as essential to know the circumstances that gave existence to a medical maxim; to understand the soil in which it grew, and to give everything its weight and proper bearing, as to know what the maxim is. When these things are forgotten, a high and honest authority may become a minister of evil, and Professor Wood is one of the last men to ask any one to follow his suggestions thoughtlessly. An unthinking and undiscriminating physician is not, in the least degree, benefitted by reading unless he learns to think and to discriminate. We know many, who, in apparent despair of improvement in the latter elements, pursue undeviatingly the course of not reading.

It is not our purpose to enter into a critical analysis of the work of Professor Wood. Upon several important points, of great value to Southern and Western practitioners, Professor Wood is clear, cogent and, in the main, correct. His chapter on malaria is full of instruction, and richly deserves the profound study of every practitioner who is not already familiar with the laws of that Protean agent, and the diversified diathesis it assumes. Fatal mistakes are often made by those who neglect the study of these important subjects.

We find much to commend in the chapters on intermittent and remittent fevers. The plan commended by Professor Wood for the management of the former is not in our judgment the best. We have tried the use of small doses of quinine, frequently repeated, and of large and decided doses given at once, and not repeated, and we greatly prefer the latter. Nor is it essential to precede, in all cases, the use of the quinine by purgative remedies. We have had too many opportunities for comparing these various plans of treating intermittent disease, to be easily deceived about anything in relation to them. As a general, nay almost an universal rule, it is perfectly safe, and judicious to give decided doses of quinine immediately after the sweating stage of a paroxysm is well established, and the patient need have no fear of any evil consequence. If there is reason to apprehend a short interval between the close of the phenomena of one paroxysm, and the commencement of the next, there is no necessity for waiting for the sweating stage. In such cases it is *best* to begin the use of the quinine in the hot stage rather than risk another paroxysm through want of enough interval.

The chapter upon remittent fever has our unqualified approbation. A physician who thinks for himself, and uses a proper degree of discrimination may, under the suggestions of this excellent chapter, grapple with remittent fever with great assurance of success.

The remarks of Professor Wood on typhoid fever are sound, judicious, and generally very correct, and we take great pleasure in commending them to medical readers.

These are the leading chapters that are likely to be attractive to Western readers, and they may resort to them with the certainty of finding good advice that will be serviceable to their patients.

Among the modifications of the third edition are revisions on the subject of epidemic cholera, and we regret that the Professor has permitted loose statements in regard to the philosophy of that disease, to creep into his work. An author capable of exercising the influence that Professor Wood may fairly claim, one, who like him is distinguished for fairness and truthfulness cannot be too careful in the statement of what purports to be facts. The uninformed, the unwary and unthinking seize upon statements made by such an authority as Professor Wood and soon learn to swear by them. A little care, some investigation, and a determination to be rigidly exact in all things, will, generally, save an author from grievous blunders. We should like to see the authority on which Professor Wood bases a portion of the following statement: "In general, its" (the cholera) "progress is arrested during the winter, to be resumed in the spring; though this is not invariably the case; for at Moscow it *prevailed* through most of the winter of 1830–'31; *the cold weather did not prevent its spreading along the Mississippi in the winter of* 1848–'49." We have marked in italics the objectionable features of this statement, and we are at a loss to know on what it rests. Cholera did not *prevail* in Moscow in the winter of 1830–'31, and cold weather did prevent "its spreading along the Mississippi in the winter of 1848–'49." When cholera "prevailed" in December, 1848, in New Orleans, Dr. Fenner says, "the weather was *oppressively* warm," the thermometer being at 84°. Between the 12th of December, 1848, and the 20th of January, 1849, there were nearly fourteen hundred deaths in New Orleans, from cholera. Before the ravages commenced the weather became oppressively hot, and while it continued of that character, the pestilence was dreadful in its ravages. The scourge was arrested by white frosts early in January, and steadily declined under the influence of cold weather. And cholera did not spread anywhere, in 1848–'49, on the banks of the Mississippi, where there was cold weather. We had many cases of cholera here in Louisville, that winter, but *every* case was brought from New Orleans. There was not an attack of cholera, nor of anything approaching it, in Louisville, in the winter of 1848–'49, among citizens who were not in New Orleans during the hot weather of December. Nor was there any more cholera anywhere on the banks of the Mississippi, during "cold weather," in 1848–'49, than there was in the year ONE. Cholera is a hot weather disease, and never originates in any other kind of weather. It may be, but that is very rare, *developed* in cold weather, but that can only occur immediately after warm weather. If, in any climate, cold weather sets in early in autumn and continues up to December, cholera will not begin in December. We defy the production of any such occurrence.

If the Professor will take the pains to examine Fenner's Southern Medical Reports, and B[illegible]el's paper in the New York Medical Journal, on the prevalence of cholera in New Orleans in the winter of 1848–'49, and its prevalence in New York in 1849, he will find the following impressive and conclusive facts on the subject:

In New Orleans, in seventeen days from the commencement of cholera, the deaths amounted to forty-five. As the weather increased in warmth, the disease spread and raged in both cities. From the 5th of December to the 16th of December the temperature gradually increased, and cholera increased slowly; from the 16th to the 22d of December the weather was oppressively warm in New Orleans, and cholera increased rapidly in its ravages, and spread itself over the city, and on the 28th reached its zenith. In the city of New York the temperature increased gradually, in May, and cholera increased gradually and slowly. Dr. Buel says: "during the first seven days of June the weather became warm, and the disease spread rapidly in all directions. Within this period seventy cases were received into the hospital, being double the whole number admitted during the whole previous time the hospital had been open. At the end of the first week in June, or about twenty-eight days from the appearance of the first case, the pestilence had become pretty extensively and generally diffused over the city. At the end of the third week in July, or about seventy-two days from its first appearance, it had arrived at its "culminating point." In New Orleans the increase of the temperature, after it began to rise, was rapid, and intense, and a corresponding state of things existed in the march of cholera. The disease in New Orleans was checked by a sudden check of the summer temperature. In New York the temperature and disease increased gradually and uniformly. Its decline depending upon the diminution of one of the elements essential to the production of the cause of cholera—moisture, was gradual and uniform, but more rapid than its increase. In New Orleans the element removed was solar heat, and as the removal was sudden, the effect was as sudden as could be expected where many had imbibed the cause without having succumbed to it, up to the time the farther production of the cause was stopped. In New York, the element removed was moisture, and that was removed more slowly than the solar heat was in New Orleans, consequently the cause ceased its operations more gradually. In the production of the cause of cholera, too much moisture plays the same part that too small an amount does.

We earnestly hope that in a future edition of this valuable work, Professor Wood will show that he has thoroughly investigated the important point we have criticised. The statement on which we have animadverted is, one of too much moment to pass unnoticed. By what possible means is the medical profession ever to arrive at the etiology of cholera, if the phenomena are continually misrepresented, or perverted by loose and unguarded statements? If the premises are false, the deductions from them must be akin to them. However much we may now despair of truthful conclusions respecting the associated circumstances of cholera, we may hope for a better future, provided that hope has a proper basis. But if the future is to improve, the present must reform. Facts must be gathered, sifted and stated accurately. Hundreds of writers might be inaccurate with less injury to the cause of truth, than one such author as Professor Wood. He is not a mere theorist; he is no hobby rider, but is recognized as a man of truth; as a philosopher beyond the suspicion of misstatements. His medical works, too, are certain to live, and we should rejoice to see them passed down to posterity without a flaw to mar their merits, without an error to injure medical science.

We cannot but regard the third edition of Wood's Practice of Medicine as the ablest contribution of its kind to American Practical Medicine. It was proper that he who fills the chair once adorned with the eloquent enthusiasm of Rush, and more recently with the eminent abilities of Chapman should do something to show that the mantle of such prophets had descended upon shoulders worthy to wear it. It has been the good fortune of Professor Wood to do more than this; he has shown that he stands among such prophets as Saul stood among the people of his day —taller from the shoulders upwards. Where Professor Rush revelled in the luxuriance of a prurient fancy, and the most preposterous theories, Professor Wood abounds in sound, practical sense, upon which men may fix their faith. Where Dr. Rush was all wrong, Dr. Wood is thoroughly right, and more sound philosophy may be deduced from any of the subjects treated by Professor Wood, than can be extracted from all the tomes of Dr. Rush. When, from the vantage ground now occupied by the profession, we look upon the labors of Dr. Rush, and recognize the vast influence he wielded, and the manner in which he lorded it over the intellects of men; we may safely congratulate alike the medical profession and humanity.

We cannot too earnestly commend the work of Professor Wood to the attention of medical readers. This third edition is worthy of a place in any medical library, no matter how full it may be, nor how choice the selection is.

The work is well printed, and does credit to the publishers. It may be purchased in this city at the book store of Morton & Griswold. B.

